# IRF3 function and immunological gaps in sepsis

**DOI:** 10.3389/fimmu.2024.1336813

**Published:** 2024-02-05

**Authors:** Bristy Basak, Sachiko Akashi-Takamura

**Affiliations:** Department of Microbiology and Immunology, School of Medicine, Aichi Medical University, Nagakute, Aichi, Japan

**Keywords:** lipopolysaccharide (LPS), TLR4 signaling, MyD88-depending pathway, IRF3 signaling, sepsis control

## Abstract

Lipopolysaccharide (LPS) induces potent cell activation via Toll-like receptor 4/myeloid differentiation protein 2 (TLR4/MD-2), often leading to septic death and cytokine storm. TLR4 signaling is diverted to the classical acute innate immune, inflammation-driving pathway in conjunction with the classical NF-κB pivot of MyD88, leading to epigenetic linkage shifts in nuclear pro-inflammatory transcription and chromatin structure-function; in addition, TLR4 signaling to the TIR domain-containing adapter-induced IFN-β (TRIF) apparatus and to nuclear pivots that signal the association of interferons alpha and beta (IFN-α and IFN-β) with acute inflammation, often coupled with oxidants favor inhibition or resistance to tissue injury. Although the immune response to LPS, which causes sepsis, has been clarified in this manner, there are still many current gaps in sepsis immunology to reduce mortality. Recently, selective agonists and inhibitors of LPS signals have been reported, and there are scattered reports on LPS tolerance and control of sepsis development. In particular, IRF3 signaling has been reported to be involved not only in sepsis but also in increased pathogen clearance associated with changes in the gut microbiota. Here, we summarize the LPS recognition system, main findings related to the IRF3, and finally immunological gaps in sepsis.

## 1 Introduction

Septic shock is characterized by a dangerous degree of hypotension, high fever, tachycardia and tachypnea due to severe sepsis (1) by various mechanisms, including the direct effects of bacterial toxins, such as endotoxin. Severe sepsis causes circulatory, cellular, and metabolic disturbances, as a result, organs are unable to receive adequate blood supply, resulting in dysfunction (2). In spite of careful treatments, the mortality rate in sepsis is still high (3). Immune-modulating therapy is the main treatment of septic shock, and extracorporeal blood purification remains contentious (2). Furthermore, it is unclear how the various innate immune pathways relate to induction or regulation of sepsis, because there are still many current gaps in sepsis immunology to reduce mortality.

Interferon regulatory factor 3 (IRF3) is a transcription regulator in many cell types, and key to rapid antiviral immune responses (4). After microbial infection, IRF3 is rapidly phosphorylated by kinases that are part of several pattern recognition receptors (PRRs) pathways such as TLR3 (5), TLR4 (6), retinoic acid–inducible gene I (RIG-I) (7), melanoma differentiation-associated protein 5 (MDA5) (8), and stimulator of IFN genes (STING) (9). After phosphorylation, IRF3 dimerizes and localizes to the nucleus, then it works as an integrating transcription factor for promoters of type I IFNs, several cytokines, as well as several antiviral IFN-stimulated genes (ISGs) (4). There are many reviews of IRF3 function during viral infection, but few recent reviews of IRF3 function in response to endotoxin. Here, we firstly review the mechanism of LPS recognition, secondly present results from IRF3-deficient mice, IRF3 inhibitors, and molecules that affect IRF3 in endotoxic sepsis, and outline the effects of IRF3 on bacterial clearance. Finally, we provide an overview of current gaps in sepsis immunology.

## 2 LPS recognition and response mechanism

LPS, also known as endotoxin, is a glycan-based pathogen-associated molecular pattern (PAMP) found on the cell surface of gram-negative bacteria that is composed of three domains: an amphipathic glycophospholipid (lipid A) component, hydrophilic polysaccharides in the core, and an O-antigen consisting of repeated units of common hexose sugar (10). Lipid A is a major inducer of the endotoxic properties of LPS. The induction of inflammatory responses by LPS starts with its binding to LPS binding protein (LBP), a 60 KD acute phase protein synthesized preliminary in the liver (11, 12). LPS aggregates in aqueous environments owing to its amphiphilic nature (13). LBP disrupts aggregation prior to its transfer to a cluster of differentiation 14 (CD14), which can be found either in a soluble form or linked to the cell surface by glycosylphosphatidylinositol anchor (14, 15).

### 2.1 TLR4 signaling diverting into the MyD88 classic NF-κB activation

The signaling responses are mainly supplied by TLR4 which is non-covalently linked to the soluble protein MD-2 (sMD-2) and secreted as a large disulfide-bound multimeric glycoprotein. CD14 immediately leaves LPS-LBP complexes after LPS is transferred to MD-2 (13). The association of the monomers LPS and MD-2 with the ectodomain of TLR4 triggers a series of consecutive events, including the dimerization of TLR4/MD-2 (16). This association triggers the formation of a hexametric ligand-receptor complex consisting of two copies of the TLR4/MD-2/LPS homodimer, an active complex. This results in the assembly of the intracellular Toll interleukin-1 receptor (TIR) domain, which further recruits downstream adaptor proteins that mediate the signaling cascade. TLR4 transduces the signals via two distinct pathways: MyD88 and TRIF dependent.

**Figure 1:**
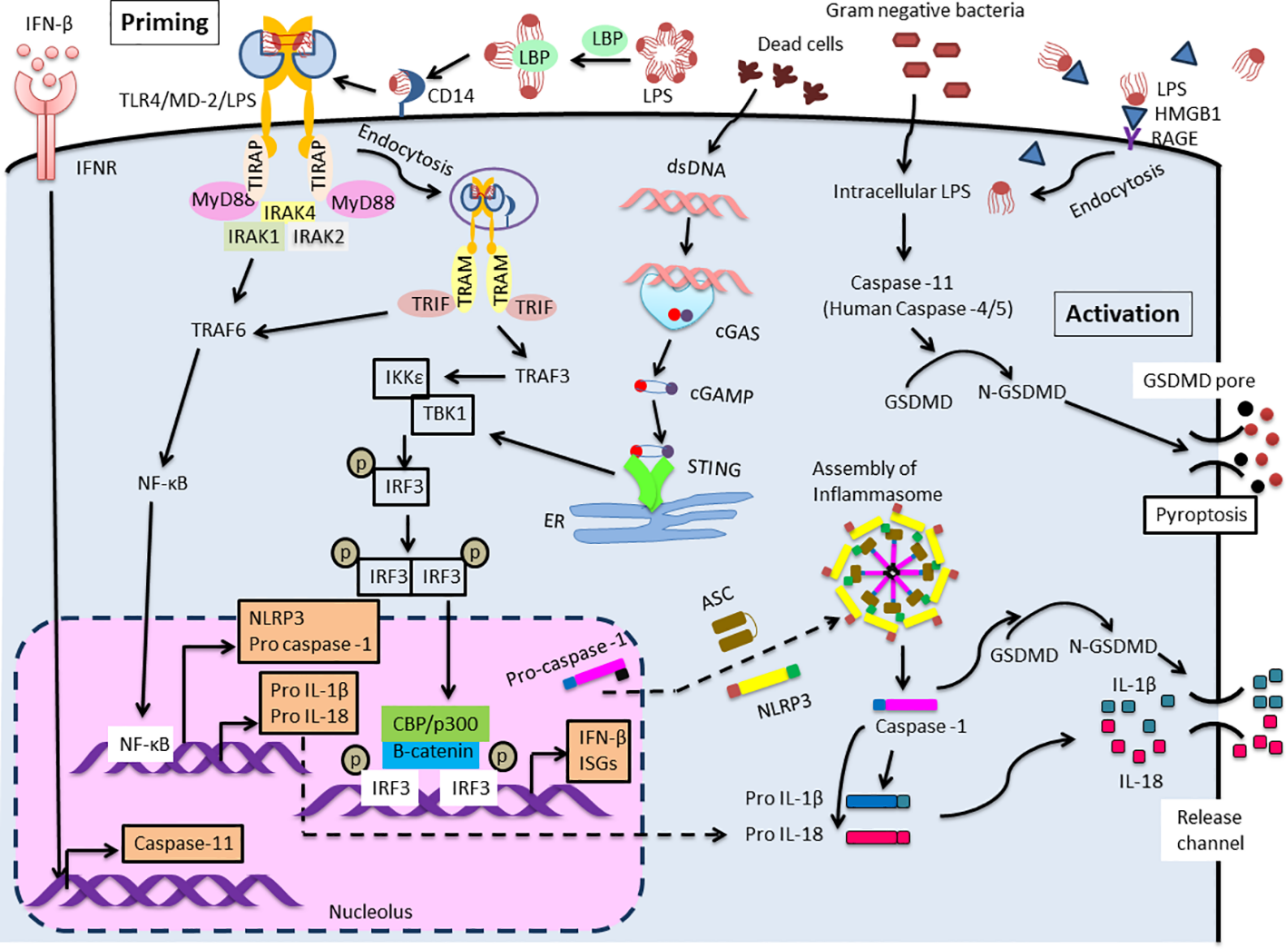
Extra- and Intra-cellular LPS recognition mechanism. TLR4 mediated NLRP3 (canonical) inflammasome signaling cascade consists of NLRP3, ASC and caspase-1 which leads to maturation and release of IL-1β and IL-18. Two distinct pathways are involved to trigger this signaling cascade. The first one is priming initiated with the recognition of extracellular LPS by TLR4. This activates NF-κB leading to pro IL-1β and pro IL-18 and up-regulates the expression of NLRP3 and pro-caspase-1. Expression of caspase-11 is also elevated by IFN-β/IFNR signaling. IRF3 and STING function as primary stimulator for IFN-β production. Another one is activation induced in response to intracellular LPS which is recognized by caspase-11 (Human: caspase-4/5). This results in the formation of non-canonical inflammasome complex and then activation, that is followed by pyroptosis through GSDMD cleavage. Meanwhile, the activation of IL-1β and IL-18 during the process of pyroptosis is mediated by processing of precursors of these cytokines by caspase-1.

The MyD88-dependent pathway commences on the plasma membrane, whereas the TRIF-mediated signaling begins after internalization of the TLR4/MD-2/LPS complex into endosomes (17). The MyD88-dependent pathway is indispensable for the production of inflammatory cytokines (18, 19). After TIRAP (also known as MAL) is recruited to the TLR4, MyD88 triggers the formation of a complex by binding to serine/threonine kinases, interleukin-1 receptor-associated kinases 2 and 4 (IRAK2 and IRAK4) (20, 21). Then, TNF receptor-associated factor 6 (TRAF6), an ubiquitin E3 ligase, is recruited and NF-κB is activated, then proinflammatory cytokines are produced (**Figure 1**, priming step).

### 2.2 TLR4 signaling diverting into the TRIF activation

A lack of MyD88 in cells might lead to the MyD88-independent pathway, initiating the late phase of the TLR4 signaling pathway (22). Additionally, CD14 mediates the internalization of the TLR4/MD-2/LPS complex into endosomes (23). Once MyD88 and TIRAP are discharged from the plasma membrane, TLR4 is triggered to harbor into the endosomes where it binds to TRIF. TRAM, also known as TICAM-2, facilitates the interaction between TRIF and TLR4 (24). Then, the E3 ubiquitin ligase TRAF3 is recruited and subsequently activates the noncanonical IKK kinases: TANK-binding kinase 1 (TBK1) and IKKϵ. TRAF3 involves in both MyD88 and the TRIF-mediated pathway, regulating both cytokine and IFN production (25). In addition, abolishing degradative (not self) TRAF3 ubiquitination inhibited all proinflammatory cytokines without affecting the IFN response. The consensus motif of TRIF is phosphorylated by TBK1 and causes recruitment of interferon regulatory factor3 (IRF3) (26). IRF3 is phosphorylated by TBK1, activated, dimerized, and translocated to the nucleus (27). IRF3 deficient mice cannot induce type 1 IFN production especially IFNβ (28). (**Figure 1**).

### 2.3 Caspase-11 activity and pyroptosis

It has recently become clear that LPS is not only recognized extracellularly via TLR4/MD-2, but also recognized intracellularly. Kayagaki et al. reported that intracellular LPS-activated caspase-11 causes cell death called pyroptosis (29). Pyroptosis is an inflammation-induced programmed cell death that involves rapid disruption of cellular membranes and the release of damage-associated molecular patterns (DAMPs). Pyroptosis is induced by caspase-1 or mouse caspase-11 (human caspase-4/5) cleaves gasdermin D (GSDMD), a pore-forming protein that is usually exists in the auto-suppressed state (30). Recently, the “tag-team” LPS recognition system, consisting of both TLR4 and caspase-11, has come to be understood as a regulator of the response to harmful infections. Extracellular LPS primarily stimulates TLR4/MD-2, followed by inducing a priming signal for the expression of inflammasome components. Intracellular LPS induces caspase-11-dependent inflammasome activation in the cytoplasm (31) (**Figure 1**, activation step). Intracellular molecules, such as caspase, or GSDMD, will be clinical target of shock therapy in the future. Further, blood LPS, and blood HMGB1, that is an important endotoxin delivery DAMPs protein and mediates caspase-11-dependent lethality in sepsis (32), may be also target for the clinical therapy.

## 3 IRF3 function in sepsis

Next, we focus on the role of IRF3 in sepsis, because IRF3 is not only an important molecule in the sepsis mortality but is also associated with important roles in microbiome.

### 3.1 IRF3 is an important gene for IFN production and cell death

IRFs are a family of transcription regulators for a wide range of genes associated with immune responses (33). Although IRF3 is expressed in almost all cells, it is localized in the cytoplasm as an inactive form. However, upon viral or duplex RNA stimulation, a specific serine residue at the C-terminus is phosphorylated, and IRF3 homodimerization was induced, then IRF3 dimer was transferred into the nucleus. After binding of β-catenin to IRF3, IRF3 also forms a complex with p300/CBP, and acquires DNA binding ability, thereby converting into the active form, subsequently increase IFN-β expression (34–37) (**Figure 1**). Further, the activation of IRF3 occurs not only through TLRs-TRIF axis, but also thorough intracellular receptors such as (RLRs)(RIG-I/MDA5)– mitochondrial antiviral signaling protein (MAVS) (38, 39) and the cytosolic DNA receptor cyclic GMP-AMP (cGAMP) synthase (cGAS)/STING axis (40). STING is a stimulator of IFN genes and the signaling pathway is activated not only by viral DNA but also by autologous genomic DNA and mitochondrial DNA through the activation of the cGAMP, then induces type I interferon, thereby relating to the promoting autophagy (41) and various kinds of cell death (e.g., apoptosis, necroptosis, pyroptosis, ferroptosis, mitotic cell death, and immunogenic cell death) (42).

### 3.2 The importance of IRF3 in endotoxin shock

IRF3 KO mice are resistant to LPS-induced endotoxin shock (28). Because these mice were found another null mutation in the *Bcl2l12* gene, Taniguchi et al. further showed that selective IRF3 deficiency in myeloid cells reduced the serum levels of type I IFN and increased the survival rate in the endotoxin-induced shock (43). Cecal ligation and puncture (CLP) model induces peritonitis by puncturing the distal part of the ligated cecum to induce leakage of feces, and this model is considered to be more clinically similar to sepsis. In the CLP model, disease score, mortality, hypothermia, and bacterial load are all reduced in IRF3 KO mice, compared to their WT counterparts (44, 45). Furthermore, in adoptive transfer experiments by using IRF3 KO bone marrow chimera, little protection from sepsis was shown, whereas chimeras with IRF3-KO stroma showed a substantial degree of protection. This result shows that IRF3 in the stroma principally enhances sepsis pathogenesis (45). From above, IRF3 functions as an immune protector through IFN production; however, IRF3 activation augments sepsis mortality.

### 3.3 Effect of IRF3 inhibitors on endotoxin shock

Several specific inhibitors of IRF3 have been reported. We focused on those that have been reported to inhibit endotoxic shock. Piceatannol (trans-3,3′,4,5′-tetrahydroxystilbene) is one of the stilbene derivative found in many plants such as red grapes. Piceatannol is known as a phytoalexin to protect the plant against fungal infection, and exhibits antioxidant and anti-inflammatory effects (46, 47). Piceatannol was previously reported as a Syk-specific tyrosine kinase inhibitor (48). Recently, the inhibitory effect on IRF3 is also known in more detail. In the nucleus, IRF3 binds to interferon stimulated response elements (ISREs) in the promoters of a subset of ISGs. Piceatannol prevents LPS-induced ISG induction by inhibiting the signaling cascade leading to IRF3 binding to the ISRE. Thus, piceatannol inhibits LPS-induced activation of IRF3, prevents the production of cytokines, and reduces septic shock after LPS challenge in animals (49). Although the reduction in mortality was limited (from >70% to < 35%), the same protective effect was also recognized using tetramethoxystilbene (TMS), a methylated form of piceatannol, instead of piceatannol. This result supports the notion that the protective effect might be related to the tyrosine kinase inhibitor, not the antioxidative properties of piceatannol (49).

Fucoxanthin (FX), which is a carotenoid derived from brown algae, binds to IRF3 and inhibits the phosphorylation of IRF3, then reduces pro-inflammatory cytokine production, thereby decreasing mortality in a mouse CLP-induced sepsis model (50). Further, FX also affects microbiota composition in intestine, that influences the development of sepsis. FX significantly reduced the bacterial load in the abdominal cavity in CLP-induced sepsis mice model via and increased the short-chain fatty acids (SCFAs) levels such as acetic and propionic acids, which showed negative correlations with the expression levels of inflammatory factors, in their intestines (51). FX alters the microbial diversity and promotes SCFAs production, in CLP-induced sepsis mice model, thus reshaping gut homeostasis (51).

The importance of IRF3 activation in the induction of septic shock is also confirmed by the results of epidermal growth factor receptor (EGFR) inhibitors (52). The binding of LPS to TLR4 and EGF to EGFR, respectively, both on the plasma membrane led to endocytosis. EGFR is important for the activation of PI3K/AKT pathway which is required for β-catenin activation and IRF-driven gene expression of TLR4 signaling. Further, the kinase activity of EGFR is needed for the activation both of IRF3 and its co-activator, β-catenin. If EGFR activity is inhibited, LPS-induced induction of IFN and ISGs is spoiled, then septic shock response is impaired. Indeed, the EGFR kinase activity inhibitor (gefitinib)-treated mice were protected from LPS-induced septic shock by the selective block of the IRF-driven genes in TLR4 signaling (52). These results demonstrate the selective regulation of TLR4 signaling by direct and indirect IRF3 inhibitors and emphasize the potential use of these inhibitors to treat sepsis.

### 3.4 Intracellular molecules and IRF3 activation

Accumulating evidence suggests that inflammation, cardiomyocyte apoptosis, and pyroptosis are involved in developing sepsis and sepsis-induced cardiomyopathy (SIC). IRF3 is important for sepsis-induced cardiac injury (53). In wild type mice and cardiomyocytes, LPS-induced cardiac injury stimulates STING through cGAS -cGAMP, which further mobilizes TBK1, then IRF3 is phosphorylated. Phosphorylated IRF3 subsequently translocated into nucleus and increased the expression of NOD-like receptor protein 3 (NLRP3). STING knockout attenuates LPS-induced cardiac injury by preventing NLRP3-mediated inflammation, apoptosis, and pyroptosis. This study suggests that STING-IRF3 might be a therapeutic target for SIC (53).

Furthermore, caspase-11 activation can also be affected by other proteins (54). The caspase-11 inflammasome in macrophages is negatively regulated by the zinc (Zn2+)-regulating protein, metallothionein 3 (MT3). In challenge with intracellular lipopolysaccharide, macrophages increased MT3 expression, which reduced the activation of caspase-11, caspase-1 and IL-1β. It is considered that MT3 increased intra-macrophage Zn2+ to downmodulate the TRIF-IRF3-STAT1 axis, a prerequisite for caspase-11 effector function. MT3 suppresses the activation of the caspase-11 inflammasome *in vivo*, whereas caspase-11 and MT3 synergistically impair antibacterial immunity (54). Thus, the inflammasome activation signal is influenced by various factors.

### 3.5 The relationship between intestinal microbiota and IRF3

As mentioned above, the CLP model is mainly used because it closely mimics the clinical progression of sepsis in humans (44). In the CLP model, bacteria, fungi, and metabolites migrate into the abdominal cavity, leading to abdominal infections and systemic sepsis. The gut microbiome plays an important role for keeping the body homeostasis, pathogen defense, food digestion and absorption, and immune system regulation (55). Preclinical studies show that microbiome-dependent metabolic pathways are able to drive immunological response against invading pathogens. Microbiome is also related to the onset of sepsis (56).

Recently, the relationship between intestinal microbiota and IRF3 has received much attention. Although the ligands are unknown, IRF3 is activated in macrophage cultures with live or sonicated commensal bacteria (44). Also, IRF3 enhances pathogen clearance by restoring host immunity. Fecal microbiota transplantation (FMT) rescued mice from lethal infection due to a pathogen community, isolated from a sepsis patient by restoring systemic immunity in an IRF3-dependent manner (57). Pathogenic community infection reduces fecal butyrate levels, which normalizes IRF3 levels. FMT, by providing operational taxonomic units (OTUs) such as Bacteroides, which can produce butyrate, and increase the expression level of IRF3, then protects against systemic pathogen community infections (57). In sepsis models, such as CLP, IRF3 functions as an enhancer of sepsis. In contrast, in pathogenic community infections, IRF3 restores the protective immunity in sepsis.

## 4 Important gaps in sepsis immunology

Even with the above mechanisms of LPS recognition as a trigger for sepsis, a variety of events, as shown in **Table 1**, actually play a significant role in the pathogenesis of sepsis.

**Table 1:** Important gaps in sepsis immunology (No mark: known, *: unknown).

1) mixing LPS responses with sepsis variation in temporal programming responses
Sepsis remains a highly heterogeneous syndrome. The immune system is changed from homeostasis in two opposite directions (excessive inflammation and immune suppression), which the various extent is dependent on individuals (58).
In sepsis patients, anti-inflammatory reactions are recognized with the pro-inflammatory response (The increase of IL-10 correlated with the rise of TNF-alpha, IL-6, and IL-8.) (59).
* Lack of insights into the direction and time course of the host response before the clinical recognition of severe disease (59)
* Lack of suitable immunological profiles which facilitates the stratification of patients with sepsis into subgroups depend on the level of severity of sepsis (58)
2) coupling to immunometabolic paralysis, as well as activation deactivation axes
Septic plasma typically exhibits a mixed environment of hyperinflammatory and immunosuppressive properties, therefore this may possibly modulate circulating leukocytes in a variety of ways. However, the evaluation of its plasma status and the regulation of circulating leukocytes are difficult, actually (59).
* Lack of suitable biomarker for the stratification of patients with sepsis into more homogeneous subgroups (58)
* Lack of research about the long-term sequelae and etiology for the development of therapy and usage as outcome parameter in clinical trials (58)
3) pathway connection with redox states of oxidation and reduction normality or excesses
Activated Pathogen recognition receptors such as TLRs increases reactive oxygen species (ROS) through NADPH oxidase enzymes and mitochondria. ROS is required for the release of pro-inflammatory cytokines (60).
Mitochondrial ROS (MtROS)-induced priming leads to de-ubiquitination of NLRP3 inflammasome, which is suggesting a non-transcriptional priming (61).
Excessive ROS due to disruption of electron transfer chain, Ca2+ overload, and depletion of endogenous antioxidants also cause cell death, including apoptosis and autophagy (62) (63). Upon mitochondrial damage, highly expressed mtROS trigger the opening of mitochondrial permeability transition pores (mPTPs) and promote apoptosis (64).
During sepsis, activated caspase-1 interacts with molecular events which promotes mitochondrial dysfunction, such as ROS production of mitochondria, disturbance of membrane permeability, and fragmentation of mitochondrial network, then exacerbates apoptosis with pro-inflammatory response (65). Apoptotic events orchestrated by elevated intracellular oxidative stress was suggested for pathogenesis of sepsis- induced acute respiratory distress syndrome (66).
Endogeneous nitric oxide (NO) has been identified as a negative regulator for NLRP inflammasome in macrophage, and enhances survival via inducing autophagy (67).
A shift from oxidative phosphorylation to glycolysis (Warburg effect) induces succinate accumulation, then the stability of hypoxia-inducible factor 1α (HIF1α) is increased, in turns increases the transcription of IL-1β (which encodes IL-1β) (68).
Increase of mitochondrial oxidation of succinate (through succinate dehydrogenase) and upregulation of mitochondrial membrane potential induce the production of mtROS, as a result, pro-inflammatory gene expression is raised (69).
* The types of mitochondrial dysfunction might have consequences on the inflammatory nature of the ongoing cell death, regulated by formulation of apoptosome or inflammasome in a context-dependent manner (70).
4) differential tissue and organ responses
In sepsis, blood and spleen leukocytes go to hyporesponsive in the acute disease stage, such as tolerance in these hematopoietic compartments. However, the functionality of alveolar macrophages, liver kupffer cells, intestinal epithelial lymphocytes, microglia cells, and skin’s CD8 T-cells was shown to be unaffected or primed (71) (72). Thus, compartments other than the blood participate in shaping immunosuppression.
* Understanding organ-specific immune responses to sepsis is important for the recognition how the local control of the infection affect the whole immunity and progression of sepsis, persistent inflammation, immunosuppression, and catabolism syndrome (59).
5) cell metabolism that provides nutrient fueling or depletion into starvation and the reversing to regaining cell satiety/homeostasis
Malnutrition can negatively affect immunity and increase the frequency of sepsis (73).
Zinc is essential in resistance against sepsis because of the modulatory effect on the inflammatory response, phagocytosis, chemotaxis, and oxidative stress (74).
Vitamin D is indispensable role in the function of innate immunity, and low serum concentration of 25-hydroxyvitamin D is related with an increase of long-term risk of subsequent community-acquired sepsis (75).
Significant switch of metabolic state (i.e.elevated saturated fatty acid, absence of ketogenesis) during sepsis goes to inflammasome signaling and was related to the increase of lethality (70).
Autophagy is a system that maintains cellular homeostasis by removing defective intracellular proteins and organelles. In overnutrition, autophagy is reduced and mitochondrial metabolic function is impaired, leading to a worse prognosis in sepsis and ICU admission (76).
* Chronic alteration conditions, such as aging, diabetes, obesity, and the chronic use of immunosuppressors, impair the immune system's ability against infection and induce a lasting inflammation and metabolic dysfunction (59).
6) Epigenetics of sepsis
Epigenetic mechanisms are likely central to the pathogenesis of sepsis because it associates with the host-pathogen interaction (histone modification), the pathogenesis of the early pro-inflammatory response (DNA methylation), and the establishment of endotoxin tolerance (DNA methylation) (77).
In human monocytic cell lines, stimulation with LPS decreased methylation of the TNF promoter, which in turn displaced nucleosomes from the NF-κB binding site (59), then NF-κB was able to bind to the TNF promoter, resulting in upregulation of TNF transcription (77).
* Lack of human studies in the area of epigenetics, should be a priority for sepsis researchers (77)
7) Impact of microbiota on immunity
Dysbiosis in the gut has been linked to the subsequent hospitalization of patients with sepsis, and this suggests an association between the composition of the gut microbiota and sepsis susceptibility (78).
Alveolar macrophages from microbiota-depleted mice show both a reduced responsiveness to microbial stimulation and a phagocytic capacity (79).
Neutrophil from microbiota-depleted mice reduced ability to migrate into tissues in response to inflammatory signals (80).
* There are many unknowns about how the host's microbiota affects immunity (59).
* Lack of data showing the relationship between changes in microbiota before and after sepsis and the development of immune paralysis (59)
* Regulation of the microbiome has great potential for anti-septicemia in prevention and personalized treatment. Therefore, it should be elucidated how microbiota disruption creates, exacerbates, and sustains septic immune abnormalities (59).

Sepsis is a highly heterogeneous syndrome, which contains not only excessive inflammation but also immunosuppressive events. This makes it difficult to understand the status of the host response and to consider the suitable treatment strategy. Flow cytometry readouts such as monocyte HLA-DR and PD-L1 expression may be useful for the monitoring marker (58), however highly sensitive biomarker to determine the severity of sepsis even in mixture immunological situation is needed.

Nitric oxide (NO) and reactive oxygen species (ROS) play important roles in nonspecific infection defense mechanisms. The excess NO produced by inducible NO synthase (iNOS) induction reacts with ROS at the site of infection to form chemically reactive active NO species such as peroxynitrite. It exhibits strong antibacterial activity, and plays an important role in innate immunity and infection defense functions by phagocytic cells such as macrophages. On the other hand, NO and ROS damage host cells and tissues and cause oxidative stress. For the reason, the function of NO and ROS are considered as a “double-edged sword” (60).

Nutrition and cell metabolism also highly affects immunity. As shown in **Table 1**, both malnutrition and overnutrition are negatively affect immunity. Zinc, one of the micronutrients, is important for the modulatory effect in sepsis inflammation. As mentioned above, Zinc also acts the indirect inhibitor on TRIF-IRF3 activation signals (54). Metabolic state is also strongly related to the inflammasome signaling and lethality.

“Epigenetics” is a regulatory mechanism of gene expression without changes in DNA sequence, and consists of DNA methylation, histone modifications, and non-coding RNAs (77). Host inflammatory response through MyD88-dependent pathway is also characterized by epigenetic modifications in key regulatory genes, such as TNF. In human monocytic cell lines, stimulation with LPS decreased methylation of the TNF promoter, which in turn displaced nucleosomes from the NF-κB binding site, then NF-κB was able to bind to the TNF promoter, resulting in upregulation of TNF transcription (77, 81). Epigenetic mechanisms are likely central to the pathogenesis of sepsis because it associates with the host-pathogen interaction (histone modification), the pathogenesis of the early pro-inflammatory response (DNA methylation), and the establishment of endotoxin tolerance (DNA methylation) (77).

Microbiota is also important for immunity. The gut microbiome has a protective role in sepsis by keeping the gut barrier, regulating leukocyte function, and modulating immunity (82). Recently, it was reported that microbiome-dependent metabolic pathways can drive distinct immune response to pathogens (82). Other study showed that sepsis patient is characterized by a loss of diversity, lower number of the key commensal genera, and overgrowth of pathogens (82, 83). Further, Probiotics and Fecal transplantation is useful for the recolonization of the gut and for decreasing sepsis incidence and mortality (84).

## 5 Conclusion

IRF3 activation is important for the mortality both in challenged with endotoxin-induced shock and in CLP model. Recent clinical targets for endotoxic shock therapy are becoming intracellular molecules and intracellularly incorporated LPS. However, IRF3 is also important and may be one of the therapeutic targets because it affects not only the sepsis induction but also the pathogen clearance via the restoration of host immunity.

One of the features that distinguishes sepsis from uncomplicated infections is the dysregulation of the host response. Another is that at the clinical diagnosis of sepsis, not only an inflammatory response but also an immunosuppression has already occurred. Thus, immunodeficiency indirectly contributes to the perpetuation of organ failure. These two characteristics are also responsible for the difficulty of sepsis treatment (59). Further, as shown in **Table 1**, there are still gaps to be addressed. Addressing these gaps should help to better understand the physiopathology of sepsis and provide translational opportunities to improve its prevention, diagnosis, and treatment.

## Author contributions

BB: Writing – original draft. SA: Writing – review & editing.
